# Planning stereoelectroencephalography using automated lesion detection: Retrospective feasibility study

**DOI:** 10.1111/epi.16574

**Published:** 2020-06-13

**Authors:** Konrad Wagstyl, Sophie Adler, Birgit Pimpel, Aswin Chari, Kiran Seunarine, Sara Lorio, Rachel Thornton, Torsten Baldeweg, Martin Tisdall

**Affiliations:** ^1^ Wellcome Centre for Human Neuroimaging University College London London UK; ^2^ Developmental Neurosciences Great Ormond Street Institute of Child Health University College London London UK; ^3^ Great Ormond Street Hospital London UK

**Keywords:** deep learning, epilepsy, neuroimaging, pediatric, stereoelectroencephalography

## Abstract

**Objective:**

This retrospective, cross‐sectional study evaluated the feasibility and potential benefits of incorporating deep‐learning on structural magnetic resonance imaging (MRI) into planning stereoelectroencephalography (sEEG) implantation in pediatric patients with diagnostically complex drug‐resistant epilepsy. This study aimed to assess the degree of colocalization between automated lesion detection and the seizure onset zone (SOZ) as assessed by sEEG.

**Methods:**

A neural network classifier was applied to cortical features from MRI data from three cohorts. (1) The network was trained and cross‐validated using 34 patients with visible focal cortical dysplasias (FCDs). (2) Specificity was assessed in 20 pediatric healthy controls. (3) Feasibility of incorporation into sEEG implantation plans was evaluated in 34 sEEG patients. Coordinates of sEEG contacts were coregistered with classifier‐predicted lesions. sEEG contacts in seizure onset and irritative tissue were identified by clinical neurophysiologists. A distance of <10 mm between SOZ contacts and classifier‐predicted lesions was considered colocalization.

**Results:**

In patients with radiologically defined lesions, classifier sensitivity was 74% (25/34 lesions detected). No clusters were detected in the controls (specificity = 100%). Of the total 34 sEEG patients, 21 patients had a focal cortical SOZ, of whom eight were histopathologically confirmed as having an FCD. The algorithm correctly detected seven of eight of these FCDs (86%). In patients with histopathologically heterogeneous focal cortical lesions, there was colocalization between classifier output and SOZ contacts in 62%. In three patients, the electroclinical profile was indicative of focal epilepsy, but no SOZ was localized on sEEG. In these patients, the classifier identified additional abnormalities that had not been implanted.

**Significance:**

There was a high degree of colocalization between automated lesion detection and sEEG. We have created a framework for incorporation of deep‐learning–based MRI lesion detection into sEEG implantation planning. Our findings support the prospective evaluation of automated MRI analysis to plan optimal electrode trajectories.

**Key Points**

- A machine‐learning classifier was successfully trained to identify FCDs
- Results were concordant with sEEG seizure onset zone in 62% of focal epilepsies and 86% of histopathologically confirmed FCDs
- Additional unimplanted abnormalities were detected in patients with focal epilepsy but where seizure onset zone was not implanted
- Automated MRI analysis involving machine learning may assist planning of sEEG electrode trajectories

## 1 INTRODUCTION

One‐third of children with epilepsy are medication‐resistant.^1^ In children with a focal seizure onset zone (SOZ), neurosurgical resection can offer seizure freedom in approximately 70%.^2^ Surgical treatment is planned by the multidisciplinary team (MDT) considering results from seizure semiology; neuropsychological, neurodevelopmental, and neuropsychiatric evaluation; and noninvasive neuroimaging techniques, including video‐electroencephalographic telemetry, magnetic resonance imaging (MRI), positron emission tomography, and magnetoencephalography. In complex patients, these noninvasive investigations can be inconclusive.

Stereoelectroencephalography (sEEG) can be used to delineate the SOZ in complex patients.^3^ In this procedure, implanted depth electrodes directly record brain activity. Currently, electrode placement is a clinical decision based on hypotheses generated by the MDT. In half of the patients selected for sEEG, the MRI scan does not show any lesions or shows nonspecific abnormalities,^4^ limiting the ability to accurately target potential areas of seizure onset.

Using machine learning, automated lesion detection methods aim to generate putative lesion locations based on structural MRIs. These approaches include voxel‐based methods^5^, ^6^, ^7^ and surface‐based approaches.^8^ We have previously developed an openly available, robust, and replicated surface‐based method to identify focal cortical dysplasias (FCDs).^9^, ^10^, ^11^, ^12^ However, most previous studies were based on cohorts with histologically or radiologically confirmed FCDs, which do not fully capture the complexity and heterogeneity of diagnostically inconclusive patients who present for presurgical evaluation by the MDT.

This retrospective study aimed to create and evaluate a framework for informing and adjusting sEEG electrode planning using automated lesion detection. We trained a classifier to detect focal cortical lesions in patients with MRI‐positive FCDs and evaluated it on complex patients who had undergone sEEG. Classifier‐identified clusters were coregistered to sEEG electrodes and were assessed for colocalization with the SOZ.

## 2 MATERIALS AND METHODS

### 2.1 Participants

#### 2.1.1 MRI‐positive cohort

A retrospective cohort of 34 patients (mean age = 11.6 years, range = 3.6‐18.5, female = 20) from Great Ormond Street Hospital (GOSH) was studied, following permission by the hospital ethical review board. Patients were included if they had a radiologically identified FCD and underwent three‐dimensional (3D) T1‐weighted (T1w) and fluid‐attenuated inversion recovery (FLAIR) imaging on the 3‐T MRI scanner at GOSH. Patients younger than 3 years of age, with MRI scans showing severe motion artifacts (ie, indistinguishable adjacent gyri due to motion or severe ringing), or without the full protocol described in the following section were excluded.

#### 2.1.2 sEEG cohort

**FIGURE 1:**
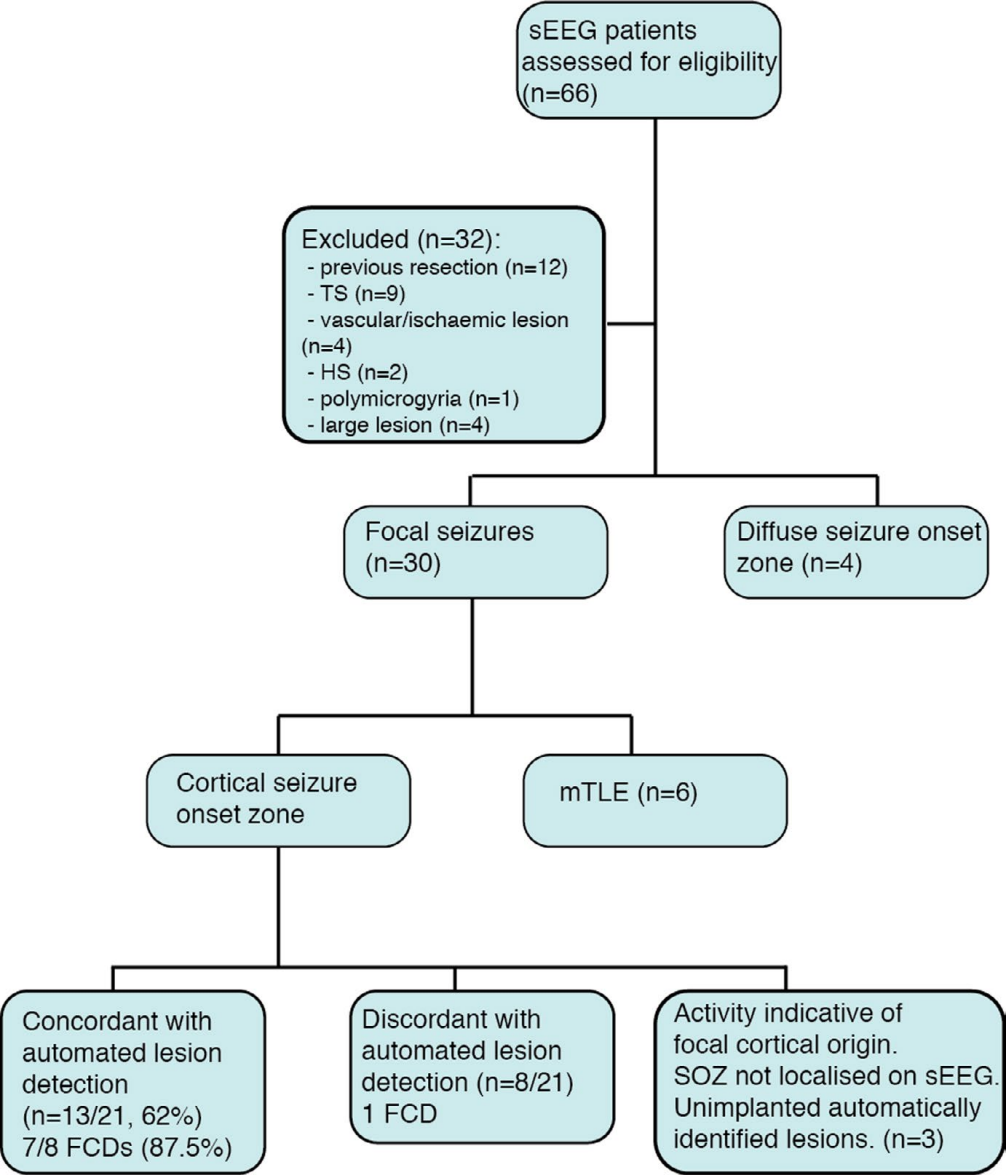
Flowchart of inclusion criteria and magnetic resonance imaging/stereoelectroencephalography (sEEG) colocalization results. Flowchart of inclusion criteria, sEEG results, and concordance with automated lesion detection in patients who underwent sEEG are shown. FCD, focal cortical dysplasia; HS, hippocampal sclerosis; mTLE, mesial temporal lobe epilepsy; SOZ, seizure onset zone; TS, tuberous sclerosis

All patients who underwent sEEG at GOSH between 2015 and 2018 were identified (n = 66; Figure 1). sEEG patients were excluded for the following reasons: radiological diagnosis of tuberous sclerosis (n = 9), hippocampal sclerosis (n = 2), vascular/ischemic lesion (n = 4), polymicrogyria (n = 1), previous resection (n = 12), and large MRI‐detectable lesions where the indication for sEEG was to determine lesion extent (n = 4). The final number of sEEG patients was 34 (mean age = 11.7 years, range = 3.6‐18.5, female = 17).

#### 2.1.3 Healthy controls

A control group of 20 term‐born participants (mean age = 16.8 years, range = 8.4‐28.2, female = 14) with no history of any neurological diagnosis was included.

#### 2.1.4 MRI acquisition

All patients and controls were scanned on a 3‐T whole‐body MRI system (Magnetom Prisma, Siemens Medical Systems), using a 20‐channel receive head coil and body coil for transmission and 80‐mT/m magnetic field gradients. 3D structural T1w images and FLAIR images were acquired using the following protocols: magnetization‐prepared rapid acquisition gradient echo (repetition time [TR] = 2300 milliseconds, echo time [TE] = 2.74 milliseconds, field of view [FOV] = 256 × 256 mm, flip angle = 8°, voxel size = 1×1 × 1 mm^3^) and FLAIR (TR = 4000 milliseconds, TE = 395 milliseconds, inversion time = 1800 milliseconds, FOV = 256 × 256 mm, flip angle = 120°, voxel size = 0.65 × 1 × 0.65 mm^3^).

#### 2.1.5 MRI postprocessing

**FIGURE 2:**
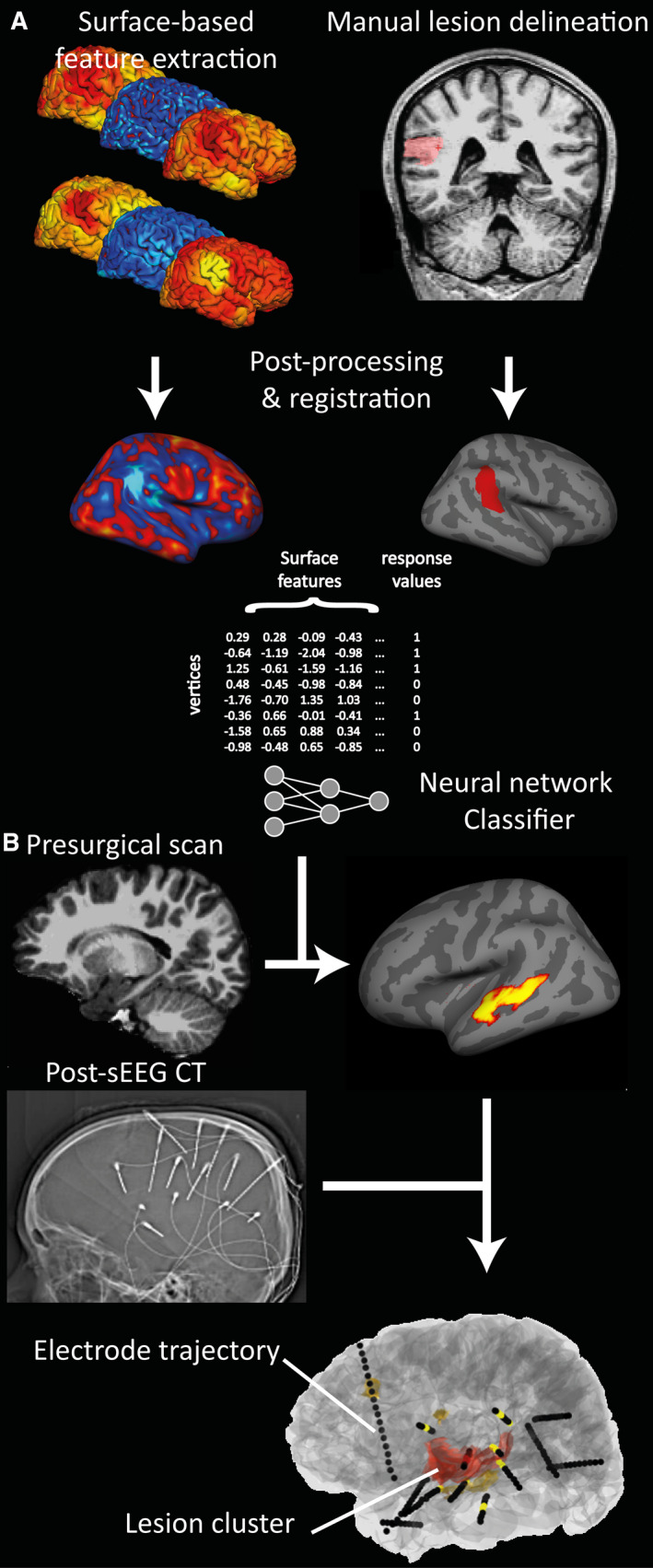
Pipeline for automated lesion detection and colocalization with stereoelectroencephalography (sEEG) electrodes. A, Surface‐based feature extraction, lesion labeling, and training of neural network classifier on magnetic resonance imaging (MRI)‐positive patient cohort. B, Testing of classifier on presurgical MRI of patients undergoing sEEG. Coregistration of classifier output clusters with sEEG electrode implantations extracted from postsurgical computed tomography (CT) is shown

Surface‐based postprocessing of T1 and FLAIR data followed our previously published automated FCD detection pipeline (https://github.com/kwagstyl/FCDdetection; Figure 2).^9^ In brief:

1. Cortical reconstructions were generated using FreeSurfer version 5.3^13^ for all participants. This generates gray and white matter triangulated mesh surfaces, where vertices are paired between the surfaces.
2. Lesion masks were created for the 34 MRI‐positive patients. Focal cortical lesions were identified on T1w and FLAIR images by an experienced pediatric neuroradiologist. 3D binary masks were manually delineated. The lesion masks were first mapped onto the individual surface reconstructions and then onto the bilaterally symmetric template (*fsaverage_sym*).^14^
3. Measures of morphological/intensity features. The following measures were calculated per vertex across the cortical surface in all participants: (a) cortical thickness, (b) intensity at the gray‐white matter contrast, (c) curvature, (d) sulcal depth, (e) intrinsic curvature, and (f) FLAIR signal intensity sampled at 25%, 50%, and 75% of the cortical thickness as well as at the gray‐white matter boundary and 0.5 and 1 mm subcortically. The choice of features was motivated by the clinical features radiologists use to identify cortical lesions as well as our previous work evaluating the discriminatory power of these features.^9^
4. Smoothing. The following features were smoothed with a 10‐mm Gaussian kernel: cortical thickness, intensity at the gray‐white matter contrast, and FLAIR intensities at all cortical and subcortical depths, to increase the stability of per‐vertex measures. Intrinsic curvature was smoothed with a 20‐mm Gaussian kernel to provide a measure of folding pattern abnormalities that is stable across adjacent gyri and sulci. Manual delineated lesions had a median area of 1185 mm^2^ and median absolute deviation of 789 mm^2^, which is much larger than these smoothing kernels.
5. Registration to a bilaterally symmetrical template space. All features were registered to *fsaverage_sym*.
6. Normalization of features. Features underwent two normalization procedures. (a) Features were normalized using a within‐subject *z* scoring, which adjusts for interindividual differences in the mean and standard deviation, for example age‐related changes in cortical thickness. (b) Features were normalized using a between‐subject *z* scoring, where each participant's per‐vertex feature was normalized by the mean and standard deviation in the population of healthy controls. This adjusts for interregional differences in the mean and standard deviation, for example, normal variability in cortical thickness across the cortex.
7. Interhemispheric asymmetry. The right hemisphere vertex values for each feature were subtracted from the left hemisphere values to create a left hemisphere asymmetry map and vice versa for the right hemisphere.
8. Deep‐learning classification. The Neural Network Toolbox in MATLAB R2018a (MathWorks) was used to create a nonlinear classifier. To avoid exhaustively testing different neural network architectures, which can lead to overfitting, we used one hidden layer, and the number of nodes in this layer was determined as follows. We ran a principal component analysis on the input surface‐based features in the control cohort, and used the minimum number of principal components needed to explain >99% of the variance as the number of hidden nodes. The neural network was trained to classify each vertex as being either lesional or nonlesional. The network was trained on surface‐based measures from vertices from each MRI‐positive patient. The input measures were normalized cortical thickness, normalized gray‐white matter intensity contrast, sulcal depth, mean curvature, the six normalized FLAIR intensity samples at different cortical depths, and intrinsic curvature, as well as the interhemispheric asymmetry measures. For training, vertices within the manual lesion masks were extracted as lesional examples, and an equal number of randomly selected vertices from the contralateral nonlesional hemisphere were extracted as healthy examples.
9. Clustering and thresholding. Output per‐vertex predictions were grouped into neighbor‐connected clusters of vertices with predicted lesion values above a specified threshold. The threshold for the classifier was determined by calculating the Youden index (sensitivity + specificity − 100) on the training dataset at a range of values and identifying the optimum threshold values. Clusters smaller than 50 mm^2^ were excluded as noise.

### 2.2 Evaluation of classifier in lesion‐positive cohort and controls

To assess the accuracy of the classifier on the lesion‐positive cohort, the network was trained using a leave‐one‐out cross‐validation approach, training on 33 lesion‐positive subjects and testing on the 34th. Lesions were recorded as being detected if the predicted cluster overlapped with the manually delineated lesion mask. The network was then trained on all 34 patients and tested on the controls to calculate specificity. Any cortical clusters identified by the classifier in controls were recorded as false positives.

### 2.3 Stereoelectroencephalography

3D electrode trajectories were planned and placed within the patients’ brains using robotic‐assisted surgery.^15^ Pre‐ and postsurgical computed tomography (CT) scans were acquired along with a CT angiogram, which was coregistered to the presurgical structural T1 gadolinium‐enhanced MRI using FLIRT from FMRIB Software Library,^16^, ^17^ with a mutual information cost function. Registrations were visually inspected using Slicer (www.slicer.org).^18^ From the postsurgical CT, precise 3D coordinates were calculated for each contact along the depth electrodes (Figure 2) using an extension for semiautomated electrode contact localizer.^19^ sEEG activity was assessed by expert neurophysiologists, and individual contacts were classified as being located within the SOZ, within the irritative zone, or not involved in the epileptogenic network. The SOZ was determined by visual examination of the ictal sEEG and patterns of seizure onset identified according to published criteria.^20^, ^21^ For all patients in whom the ictal onset was considered to be focal, electrical seizure onset was before clinical onset. We also carried out stimulation studies, which produced habitual seizures in some patients, but a lack of stimulated seizure did not preclude identification of the SOZ.

### 2.4 Comparison between sEEG and MRI clusters

Lesion clusters predicted by the classifier were coregistered from the native MRI surface reconstruction to the surface reconstructions in Slicer space (Figure 2). The minimum Euclidean distance was calculated from each cluster to each electrode contact.

An automatically identified lesion cluster and sEEG were recorded as colocalized if a lesion cluster was within 10 mm of an electrode contact in the SOZ.^22^ Neuroimaging processing (K.W., S.A.) and neurophysiology assessment (B.P., R.T.) were carried out independently to avoid bias.

### 2.5 Analysis of how lesion detection would have altered electrode placement

To estimate the impact on incorporating lesion detection into prospective electrode placement, the number of additional electrodes required to sample potential lesion clusters was calculated for each patient as follows.

Predicted lesion clusters were excluded if they were:

- On the contralateral hemisphere in patients with a unilateral implantation due to strong preimplantation evidence of laterality;
- Due to an obvious artifact (eg, motion ringing or skull stripping artifacts)
- Not in the top three clusters based on classifier prediction values.

If predicted lesion clusters were already within 10 mm of an electrode contact, electrodes were classed as concordant. This threshold of 10 mm was chosen as a balance between sEEG sampling radius,^22^ the risk of two electrodes touching and impacting the quality of signal recorded,^15^ and the spatial specificity of our structural MRI features.

The number of remaining clusters that would require an additional electrode was then calculated using a rule of thumb that clusters with no electrode within 10 mm required an extra electrode to be inserted.

### 2.6 Power calculations for a prospective study

Power calculations were performed to estimate appropriate sample sizes for a future prospective study. First, we calculated the statistical likelihood of a positive contribution from our results. Then, confidence intervals for estimates of the number of positive contributions for a given sample size were generated using 1000 randomly generated cohorts where the likelihood of contributing to a patient's plan had the probability estimated from this retrospective cohort and the predicted number of positive contributions over the cohort was calculated.

### 2.7 Data and code availability

All code to replicate the automated lesion detection analyses and code to compare the automated lesion detection with sEEG depth electrode contacts are freely available from https://github.com/MELDProject. A full results table is also available from https://github.com/MELDProject. Figures were plotted using nilearn^23^ and raincloud plots.^24^

## 3 RESULTS

### 3.1 Classifier lesion detection results in MRI‐positive cohort and controls

Of the 34 patients with visible FCD on MRI, the classifier was able to detect the lesion in 25 (sensitivity = 74%). In two of the nine undetected patients, no clusters were found. In the remaining seven patients, between one and four clusters were found. In the 20 healthy controls, no clusters were detected (specificity = 100%).

### 3.2 sEEG implantation

#### 3.2.1 Indication

There were three types of indications for sEEG implantation (Table 1): (1) discordance: 13 patients were implanted where a lesion had been identified preoperatively but other data suggested the SOZ may be located elsewhere; (2) five patients were implanted because the MRI was not definitive; and (3) 16 patients were implanted because no lesion was identified on MRI (MRI‐negative).

**TABLE 1:** Table of results from comparison between automated MRI lesion detection and sEEG along with presurgical sEEG indication, and post surgical seizure‐freedom and histology

Patient	sEEG indication	sEEG outcome	Clusters, n	Concordance of sEEG & automated clusters	Surgery	Histology	Outcome	Follow‐up time, mo
1	Lesion‐negative	Focal	2	N	TC & laser	n.a.	Seizure‐free	4
2	Lesion‐negative	Focal	0	N	Y	non‐diag	Seizure‐free	27
3	Lesion‐negative	Focal	3	Y	Y	non‐diag	Not seizure‐free	8
4	Lesion‐negative	Focal	2	N	N	n.a.	n.a.	n.a.
5	Lesion‐negative	Focal	0	N	N	n.a.	n.a.	n.a.
6	Lesion‐negative	Focal	1	N	Y	FCD IIA	Seizure‐free	2
7	Discordance	Focal	4	Y	Y	FCD IIB	Seizure‐free	14
8	Discordance	Focal	7	Y	Y	non‐diag	Seizure‐free	45
9	Discordance	Focal	3	Y	Y	FCD II	Seizure‐free	22
10	Discordance	Focal	5	Y	Y	non‐diag	Not seizure‐free	14
11	Discordance	Focal	1	Y	Y	FCD IIB	Seizure‐free	28
12	Discordance	Focal	2	N	Y	Other	Not seizure‐free	18
13	Discordance	Focal	2	Y	Y	Other	Not seizure‐free	16
14	Discordance	Focal	4	Y	Y	FCD IIB	Seizure‐free	7
15	Discordance	Focal	3	N	Y	non‐diag	Not seizure‐free	7
16	Not definitive	Focal	1	Y	Y	FCD IIB	Seizure‐free	12
17	Not definitive	Focal	3	Y	Y	non‐diag	Seizure‐free	2
18	Not definitive	Focal	2	Y	Y	FCD IIB	Seizure‐free	21
19	Lesion‐negative	Focal	4	Y	N	n.a.	n.a.	n.a.
20	Discordance	Focal	6	Y	Y	FCD IIA	Seizure‐free	2
21	Discordance	Focal	1	N	TC	n.a.	Not seizure‐free	10
22	Lesion‐negative	mTLE	1	n.a.	Y	non‐diag	Seizure‐free	13
23	Lesion‐negative	mTLE	1	n.a.	Y	non‐diag	Seizure‐free	20
24	Lesion‐negative	mTLE	0	n.a.	Y	Other	Not seizure‐free	17
25	Lesion‐negative	mTLE	0	n.a.	Y	non‐diag	Not seizure‐free	14
26	Lesion‐negative	mTLE	2	n.a.	Y	non‐diag	Seizure‐free	7
27	Discordance	mTLE	1	n.a.	Y	HS	Seizure‐free	14
28	Lesion‐negative	Diffuse	1	n.a.	N	n.a.	n.a.	n.a.
29	Lesion‐negative	Diffuse	4	n.a.	N	n.a.	n.a.	n.a.
30	Lesion‐negative	Diffuse	4	n.a.	N	n.a.	n.a.	n.a.
31	Not definitive	Diffuse (Rasmussen)	1	n.a.	TC	n.a.	Not seizure‐free	23
32	Not definitive	Likely focal	2	n.a.	TC	n.a.	Not seizure‐free	28
33	Discordance	Likely focal	7	n.a.	N	n.a.	n.a.	n.a.
34	Lesion‐negative	Likely focal	6	n.a.	N	n.a.	n.a.	n.a.

Note

"Discordance" indicates that an MRI abnormality was identified but was discordant with other presurgical investigations. "Not definitive" indicates that presurgical MRI was not definitive.

Abbreviations: FCD, focal cortical dysplasia; HS, hippocampal sclerosis; MRI, magnetic resonance imaging; mTLE, mesial temporal lobe epilepsy; N, no (ie, no colocalization or no surgery); n.a., Dnot applicable; non‐diag, nondiagnostic; sEEG, stereoelectroencephalography; TC, thermocoagulation; Y, yes.

#### 3.2.2 Outcome of sEEG implantation

In 21 of 34 patients (62%), a focal cortical SOZ was identified on sEEG (Table 1). Of these 21 patients, 16 (76%) underwent subsequent epilepsy surgery and 10 of 16 (63%) were seizure‐free at last follow‐up (Engel Class 1). Histology was FCD IIB in five, FCD IIA in two, FCD II‐unspecified in one, nondiagnostic in six, and other in two patients. Of the eight patients with FCD on histology, all eight were seizure‐free at last follow‐up (Engel Class 1). Two patients underwent thermocoagulation as a therapeutic test; one responded to it and subsequently underwent laser ablation at the same location and is now seizure‐free. Three patients have not undergone resective surgery (Table 1), of whom two were offered a second sEEG implantation to more thoroughly map the SOZ and the third patient was offered thermocoagulation but the parents declined.

For seven patients, sEEG did not identify focal cortical SOZs. In three of these patients, although a focal SOZ was not identified, the pattern of seizure onset was thought to indicate a focal origin where the suspected cortical abnormality was not adequately sampled. Two of the seven patients underwent thermocoagulation. In four of the seven patients, seizure onset was described as diffuse, including one patient who has since been diagnosed with Rasmussen encephalitis.

In the final six patients, sEEG revealed a mesial temporal lobe SOZ. All six patients underwent epilepsy surgery. Histology was nondiagnostic in four patients, hippocampal sclerosis in one patient, and hippocampal gliosis in one patient. Four of six patients (67%) were seizure‐free at last follow‐up (Engel Class 1).

### 3.3 Comparison of automated lesion detection with sEEG results

**FIGURE 3:**
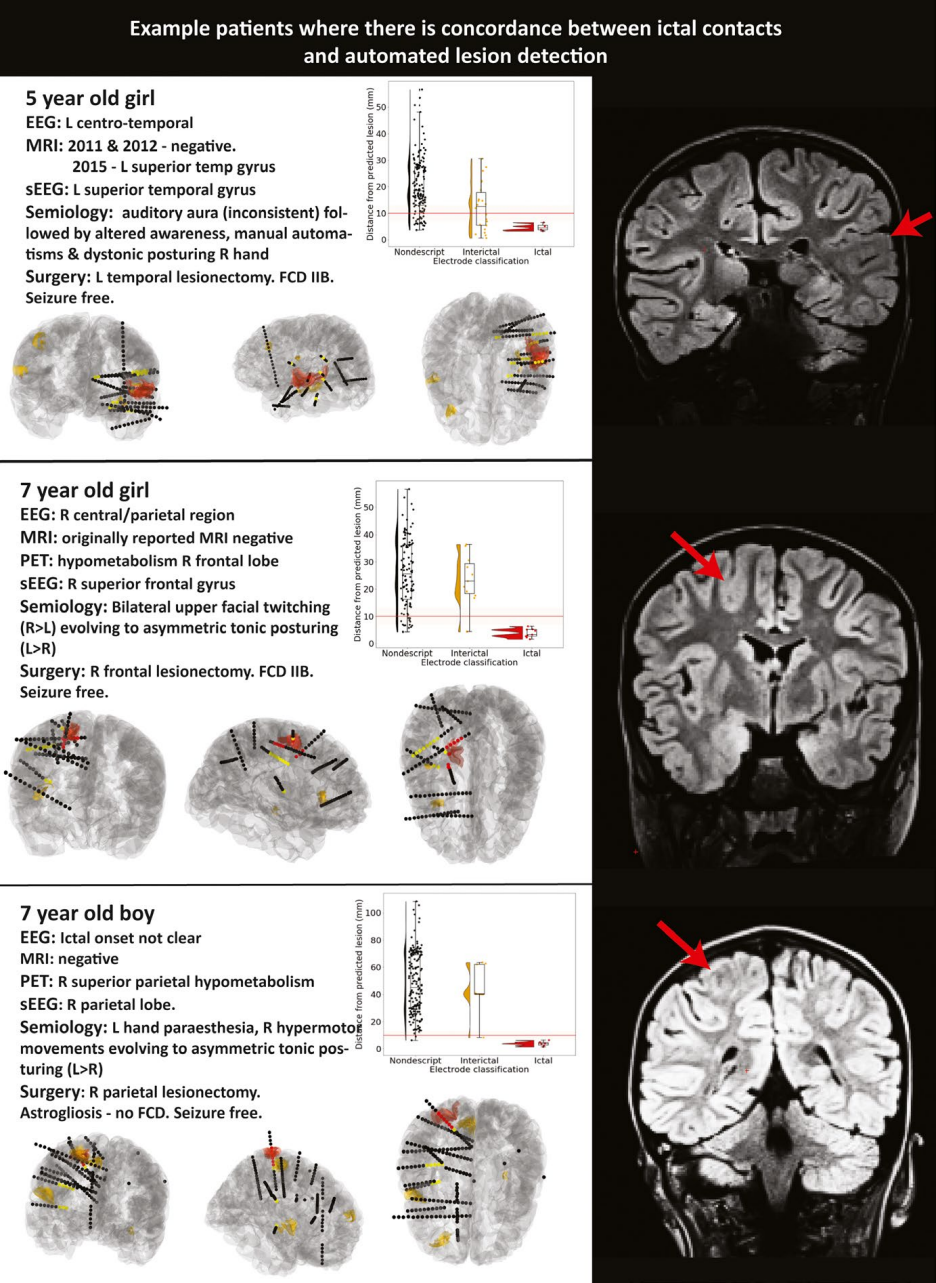
Case studies of three sample patients where there is colocalization between ictal contacts and automated lesion detection. Ictal contacts (red contacts) are within 10 mm of the automated classifier prediction (red cluster). For each patient, a brief clinical overview (left upper), a plot of distance of the stereoelectroencephalography (sEEG) contacts from the predicted lesion (right upper), visualization of the electrode positioning (ictal contacts = red, interictal = yellow, other = black) with automated clusters (red = top cluster, yellow = other clusters, lower panels), and a coronal section of fluid‐attenuated inversion recovery magnetic resonance imaging (MRI) with lesion indicated by red arrow are shown. L, left; R, right; FCD, focal cortical dysplasia; PET, positron emission tomography

Of the 21 patients in whom a focal cortical SOZ was identified on sEEG, the automatically predicted lesion was colocated with the sEEG‐determined SOZ in 13 patients (62%; Figure 1). Of the eight patients with histopathologically confirmed FCDs, the predicted lesion was colocated with the sEEG‐determined SOZ in seven patients (88%; Figure 1). Preoperatively, one of these patients was MRI‐negative, two had presurgical imaging that was not definitive, and five had discordant presurgical investigations. Of these five patients with discordance between presurgical neuroradiology and electrophysiology, three were originally MRI‐negative and subsequently had a subtle MRI abnormality suspected and the final two patients had a lesion reported on the radiology report. Three case studies where there was colocalization between the predicted lesion and the sEEG ictal contacts are presented in Figure 3.

Of the remaining 13 patients, six had mesial temporal SOZs identified by sEEG. The automated cortical analysis is unable to explicitly identify mesial temporal (ie, hippocampal and amygdala abnormalities), as these structures are not segmented as part of the cortical reconstruction. However, in two of these patients, the method did identify abnormalities in the ipsilateral temporal neocortex, consistent with findings that mesial temporal lobe epilepsy is associated with neocortical abnormalities in the ipsilateral temporal lobe.^25^ Two patients had no neocortical clusters, and two had extratemporal neocortical clusters.

There were three patients in whom the pattern of seizure onset was thought to indicate a focal origin, but where the suspected cortical abnormality was not adequately sampled. In two of these patients, our automated method identified structural cortical abnormalities on the affected hemisphere that were not implanted. It is not possible to retrospectively evaluate whether these are the epileptogenic lesions.

Across the total cohort of 34 patients, on average each patient had 2.53 ± 1.99 clusters (range = 0‐7 clusters). Interestingly, all of the six patients with focal epilepsy who were not seizure‐free postoperatively had additional neocortical clusters identified by the automated cortical analysis that were not concordant with the contacts classified as ictal on sEEG (Table 1). A future prospective study could implant these as putative lesion locations.

### 3.4 Impact on preimplantation planning

Feasibility analysis for a prospective study on using automated MRI analysis to improve detection of the SOZ in patients undergoing sEEG indicated that 14 extra electrodes would be required to ensure that the top three clusters identified by the classifier were being sampled in all 34 patients. Clusters were ranked based on their mean classifier prediction value across all vertices within each cluster. This analysis indicated that an average of one extra electrode per two sEEG patients would be required. Our power calculation indicated that we would need to evaluate a minimum of 35 patients undergoing sEEG to have 90% confidence that we could provide contributory evidence for localization of seizure foci for 10 patients. It is important to note that this cohort includes patients who would later be found to have diffuse SOZs or mesial temporal lobe epilepsy. To ensure that a concordant structural abnormality and SOZ are identified in a minimum of 10 patients, 21 patients with focal SOZs would be required. Finally, to provide a new target to implant in a patient who would otherwise not have had their SOZ detected by sEEG, we would need to implant 26 patients.

## 4 DISCUSSION

Here, we have developed a pipeline for automated detection of focal cortical lesions and tested the feasibility of incorporating this technology into planning of sEEG trajectories. In the training cohort of patients with radiologically diagnosed FCD, our classifier was able to detect 74%, while maintaining 100% specificity. In the complex cohort representative of drug‐resistant epilepsy patients who undergo sEEG at a tertiary neurosurgical center, of the eight patients who ultimately had histologically confirmed FCD type II, automated lesion detection identified lesional clusters that colocalized with SOZ electrode contacts in 88% (seven patients). Across heterogeneous histopathologies, but with focal SOZs, the automated lesion detection colocalized with SOZ contacts in 62% (13/21 patients). Incorporating automated lesion predictions into implantation strategy would require one additional electrode per two patients. Given the clinical variation in electrode numbers, the potential to identify lesional areas that might be missed, and the relatively small increase in bleeding risk of adding an extra electrode to an implantation,^26^ this work lays the framework for future prospective studies incorporating these artificial intelligence technologies into clinical practice.

In recent years, there have been considerable advances in the automated detection of focal epileptogenic abnormalities based on structural MRI scans. These approaches use a combination of postprocessing and machine‐learning techniques to automatically delineate structural abnormalities.^6^, ^8^, ^9^, ^11^ The sensitivity (74%) and specificity (100%) are in keeping with comparable surface‐based lesion detection methods with sensitivities ranging from 72% to 74% and specificities ranging from 90% to 100%.^8^, ^9^, ^11^ However, these studies generally evaluated algorithms on cohorts of histopathologically confirmed FCD cases and healthy controls, which do not reflect the complexity and heterogeneity of patients with medication‐resistant epilepsy. Therefore, it is unclear how these algorithms would perform prospectively and how they might be used to inform clinical decision‐making. This study evaluates the feasibility of these techniques on a clinically realistic complex cohort and demonstrates how such approaches could be incorporated into presurgical planning.

The advantage of incorporating these technologies is that they can provide objective lesion hypotheses even in patients in whom the SOZ is difficult to identify and may therefore generate stronger preimplantation hypotheses. For example, three patients in our study with focal seizure semiology had no SOZ identified through sEEG implantation. In two of these patients, our algorithm detected clusters that had not been implanted and were plausible lesion hypotheses. To test such hypotheses, a prospective study, where automated lesion detection is incorporated into sEEG implantation planning, is required. Positive outcomes would include increased presurgical confidence, identification of the SOZ in MRI‐negative patients, improved delineation of cortical lesion boundaries, and a potential reduction in the number of required sEEG electrodes. Such developments offer the possibility of improved clinical outcomes and reduced financial burden.

One limitation of this study is that it was retrospective. A corollary of this is that when clusters were identified that were not concordant with the SOZ, they were challenging to interpret. Across all 34 patients, the classifier detected an average of 2.53 clusters per patient. As there is incomplete sampling of the brain with sEEG, some detected MRI clusters were not close to implanted electrodes. It is not possible to determine whether these were false positives or would have exhibited ictal activity, especially in patients where histology was nondiagnostic and patients were not seizure‐free. In clusters that were inconsistent with semiology (ie clusters in patients with diffuse SOZs), it is not possible to determine whether these are false positives or whether the cortical tissue would exhibit histological abnormalities. This is further complicated by previous studies in patients with focal epilepsy^25^, ^27^, ^28^ that have identified extralesional structural abnormalities on MRI of currently unknown clinical significance.

Nevertheless, sEEG is an optimal approach for assessing any new diagnostic technique in focal epilepsy, as it is the only approach for accurate assessment of the ictal onset zone other than postoperative outcome. Thus, this validation problem is shared with any noninvasive technique, and future studies will be required to fully elucidate the electrographic and histopathological basis of these clusters.

A second limitation is the relatively small sample sizes for classifier training and validation. Future studies incorporating MRI data from focal cortical epilepsy patients and controls across multiple centers with a wide representation of ages will help to mitigate this.^29^ Additionally, larger studies will allow for the development and evaluation of deep‐learning tools such as ensemble models^30^ and graph convolutional networks.^31^

A third limitation is that we are currently restricted to detecting focal cortical lesions, and some lesion‐negative patients have mesial temporal lobe lesions (Table 1). Future development of a combined algorithm for automated cortical and mesial temporal lobe lesions is required.

In conclusion, our study demonstrates the feasibility of incorporating deep‐learning–based cortical lesion detection from structural MRI into planning of sEEG implantation in patients with suspected focal epilepsy. Additionally, we estimated the impact of implanting the extra electrodes required to adequately sample any additional automatically detected structural targets. These analyses lay the foundations for prospective evaluation of automated lesion detection in clinical practice.

## CONFLICT OF INTEREST

None of the authors has any conflict of interest to disclose. We confirm that we have read the Journal's position on issues involved in ethical publication and affirm that this report is consistent with those guidelines.

## AUTHOR CONTRIBUTIONS

Conception and design of the study: K.W., S.A., B.P., R.T., T.B., M.T. Acquisition and analysis of data: K.W., S.A., B.P., S.L., K.S., A.C., R.T., M.T. Drafting of the manuscript or figures: K.W., S.A., A.C., R.T., T.B., M.T.
